# Extracorporeal membrane oxygenation in trauma: a single-center retrospective observational study

**DOI:** 10.1007/s00068-024-02734-1

**Published:** 2025-01-27

**Authors:** Mathias Ahlqvist, Pär Forsman, Pål Morberg, Magnus Larsson, Lars Mikael Broman, Shahzad Akram

**Affiliations:** 1 ECMO Center Karolinska, Pediatric Perioperative Medicine and Intensive Care, Astrid Lindgren Children’s Hospital, Karolinska University Hospital, Akademiska straket 14, Stockholm, 17176 Sweden; 2 Department of Research and Development, Capio Saint Göran Hospital, Stockholm, Sweden; 3 Department of Acute and Trauma Surgery, Karolinska University Hospital, Stockholm, Sweden; 4 Department of Physiology and Pharmacology, Karolinska Institutet, Stockholm, Sweden; 5 Department of Anaesthesia, Vestfold Hospital Trust, Tønsberg, Norway; 6 Norwegian Air Ambulance Foundation, Oslo, Norway

**Keywords:** ECMO-Extracorporeal membrane oxygenation, VA ECMO-Veno-arterial ECMO, VV ECMO-Veno-venous ECMO, ISS-Injury severity score, NISS-New injury severity score

## Abstract

**Purpose:**

Globally, trauma is a leading cause of death in young adults. The use of extracorporeal membrane oxygenation (ECMO) in the trauma population remains controversial due to the limited published research. This study aimed to analyze 30-day survival of all the trauma ECMO patients at our center, with respect to injury severity score (ISS) and new injury severity score (NISS).

**Methods:**

We performed a retrospective analysis of all trauma patients receiving ECMO support at a Level 1 trauma center in Sweden between 1997 and 2019.

**Results:**

A total of 53 trauma patients received ECMO support. 85% were male; the median age was 24, with interquartile range (IQR) 17–44 years. More than 70% were multi-trauma patients. The mean NISS and ISS were 50 (IQR:34–57) and 42 (IQR:33–57), respectively. 62% were supported on veno-arterial ECMO with a survival benefit for veno-venous ECMO (75% vs. 36%, respectively (*p* = 0.01)). There was no association between severity in terms of trauma-score and survival. Sixteen patients (30%) were cannulated at referring hospitals and transported to our unit on ECMO with a survival of 69%, similar to those cannulated in-house. 60% of patients survived ECMO, and 51% survived to hospital discharge.

**Conclusions:**

This study indicates that trauma patients may benefit from ECMO, independent of severity. Furthermore, our results support ECMO transport as feasible in trauma patients. We recommend larger multi-center studies to determine which trauma patients would have the greatest benefit of ECMO.

**Supplementary Information:**

The online version contains supplementary material available at 10.1007/s00068-024-02734-1.

## Introduction

Globally, trauma is a leading cause of death in young adults [1]. Trauma and injuries are broadly classified as traffic accidents, falls, drowning, burns, and acts of violence against oneself or others, amongst other causes, as defined by the World Health Organisation [2]. Death within the first few hours is often related to cardiac failure, pulmonary decompensation, hemorrhage and coagulopathies [3]. In the treatment of trauma, time is thus critical. Overall, the *time-to-trauma center* is paramount to good survival outcomes [4]. The majority of traumatic injuries are blunt (90%), while penetrating injuries account for the remaining 10%. Most cases of severe multi-trauma, as defined by the American College of Surgeons Committee of Trauma Triage Criteria, present at major trauma centers [5]. To quantify the severity of trauma it is common practice to use the injury severity score (ISS) and it’s later adaptation, the new injury severity score (NISS), which shows a good correlation with survival in trauma patients [10]. The correlation between ISS and mortality is not strictly linear, as Copes et al. demonstrated, and hence the scores are usually divided into intervals [11]. Generally, an ISS of 1–8 is considered minor trauma, 9–15 moderate, 16–24 severe, and ≥ 25 very severe trauma, with a corresponding increase in risk of mortality [12].

Modern intensive care has improved in recent decades, and overall survival of patients has increased [13–14]. However, a small percentage of trauma patients do not respond to resuscitative care and may die both from acute- and chronic complications, such as shock, hypoxia, hypercapnia, and acidosis. These patients may benefit from extracorporeal life support, i.e., extracorporeal membrane oxygenation (ECMO) [15–17]. The use of ECMO in the trauma population has been described since the 1970s [18]. Yet there is no consensus, and treatment remains controversial. This is mainly because of the limited published research on ECMO in trauma, with mostly small cohort studies and a handful of reviews, as well as the limited availability of ECMO, in addition to some of the unique challenges in the severely traumatized patient [19]. Balancing anticoagulation and resuscitative surgical procedures (damage control surgery), technical difficulties in cannulation sites and vigilant monitoring for rebleeds makes the trauma patient on ECMO an especially challenging intensive care patient. ECMO indications also vary, from acute hemorrhagic shock and severe pulmonary contusions to secondary ALI or ARDS [20–21]. Recent ECMO advances, such as development of circuits, coating materials, and a broader understanding of the physiology in the trauma patient, have increased its use as an advanced critical care treatment modality [22].

The primary aim of this study was to describe the 30-day survival of patients admitted under the trauma team activation (TTA) and who were offered ECMO by our team. This includes both primary trauma patients and secondary transfer patients from other centers, as well trauma patients presenting to other centers who were cannulated and brought back to our center. Secondary aims were to describe indications, patient demographics, time to ECMO support, complications during treatment, and to compare outcome in terms of survival to the trauma severity score models. We hypothesize that, similar to previous findings, survival in the trauma population will be similar to the ECMO population as a whole.

## Methods

We performed a single center retrospective cohort analysis of both pediatric and adult trauma patients receiving ECMO at our center between 1997 and 2019. Ethical approval was obtained from the Swedish Ethical Review Authority (EPM DNR 2019–03808).

The data was collected from a Level 1 trauma center with a high volume of both trauma and ECMO patients. The center has more than 25 years of experience treating patients with pulmonary and/or cardiac pathologies across all age groups. Furthermore, the clinic has a mobile ECMO team that usually cannulates the patient at a referring hospital and then transports them back to the ECMO center, as mentioned above.

Inclusion criteria were patients who were admitted to the hospital under the trauma team activation (TTA) and who were offered ECMO, as well as trauma patients who were accepted from other hospitals. Patients who were deemed dead-on-arrival or did not survive in the trauma room were excluded. Data was collected from the hospital’s trauma registry and the department’s ECMO intensive care unit (ICU) quality database. Data was cross-checked and verified against the department’s patient medical charts, which are linked to the National Population Register, allowing for long-term follow-up of mortality. After the completion of the dataset, data was anonymized. Analysis was performed on aggregates. Patients were subdivided into three groups: early cannulation (< 12 h from the time of trauma), intermediate cannulation (12–24 h), and late cannulation (> 24 h). Patient demographics, diagnoses, ECMO mode (veno-arterial (VA) and veno-venous (VV) ECMO), type of trauma, and trauma scores (ISS and NISS) were also included in the data [23–24]. Acute lung injury and ARDS were defined according to Bernard et al. as “acute onset of diffuse bilateral pulmonary infiltrates by chest radiograph; a PaO2/FiO2 ≤ 300 for ALI and ≤ 200 for ARDS; and a pulmonary artery wedge pressure ≤ 18 or no clinical evidence of left atrial hypertension” [25]. Additionally, baseline characteristics, before (pre) and after (post) cannulation, physiologic data, complications, and outcomes were recorded. Survival was categorized by decannulation alive from ECMO and survival to hospital discharge. Survival in the Swedish National Trauma Registry was based on a 30-day survival. Injury coding in the national registry was performed manually according to the Abbreviated Injury Scale (AIS) code manual based on injuries at the time of presentation to hospital. Using the AIS (2005/2008), the ISS and NISS were calculated [26]. The scores were calculated after patient demise or hospital discharge. Furthermore, from the ISS and NISS, patients were divided into 5 groups of trauma-severity, according to Copes et al. [11].

### Statistics

Data is presented as numbers (n) and percentages (%). Normality was tested by Shapiro-Wilk’s test. Normally distributed data is presented as mean (± SD). Non-parametric data is presented as median (IQR 25-75%). Statistical analysis was performed using Mann Whitney U test. For categorical variables Fisher’s exact test was used. A p-value < 0.05 was considered statistically significant. All statistical analyses were performed using RStudio 2021.09.0.

## Results

### Pre-ECMO: demographics

**Fig. 1:**
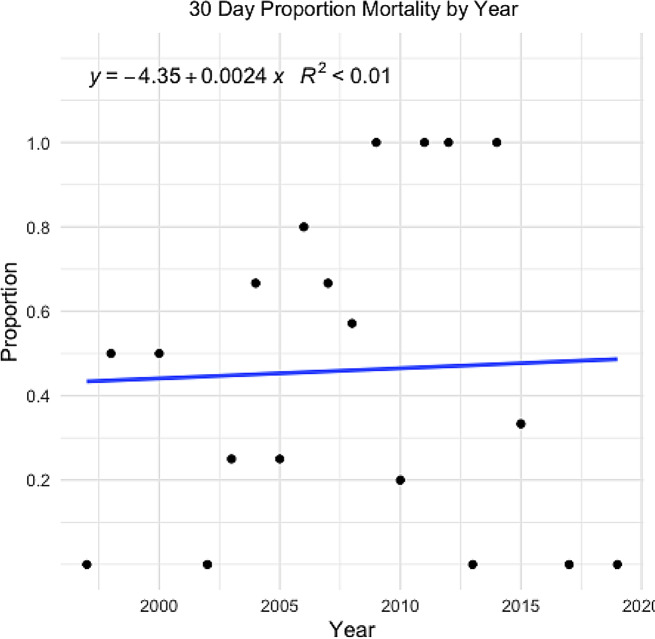
Scatter plot of the proportion of patients surviving treatment based on the year that they were admitted. A linear regression model is also displayed

During the study period 53 trauma patients received ECMO support. The majority of the patients were males (*n* = 45, 85%). The most common cause of trauma was traffic accidents, followed by falls, and burns (Table 1; Fig. 1). Over half of the patients (72%), were classified as multi-trauma, followed by severe burns, and penetrating trauma (Table 1). In three cases head injuries were the only presenting trauma; however, these patients also suffered from cardiac arrest (*n* = 2), or sepsis (*n* = 1) and were placed on ECMO due to ARDS, cardiac failure, and cardiopulmonary failure, respectively (Table 2). In total 26 patients had either an isolated head injury or a head injury in combination with multi-trauma. The overall ISS was 42 (IQR: 33–57), and NISS 50 (IQR: 34–57). Using the Copes et al. intervals of ISS, most patients had a score either between 25 and 40 or 50–65 (Table 3); there was a similar pattern for the NISS interval-scores, however, more patients were classified as being in the two upper intervals, i.e. 50–65 and 66–75 (Table 4).

**Table 1:**
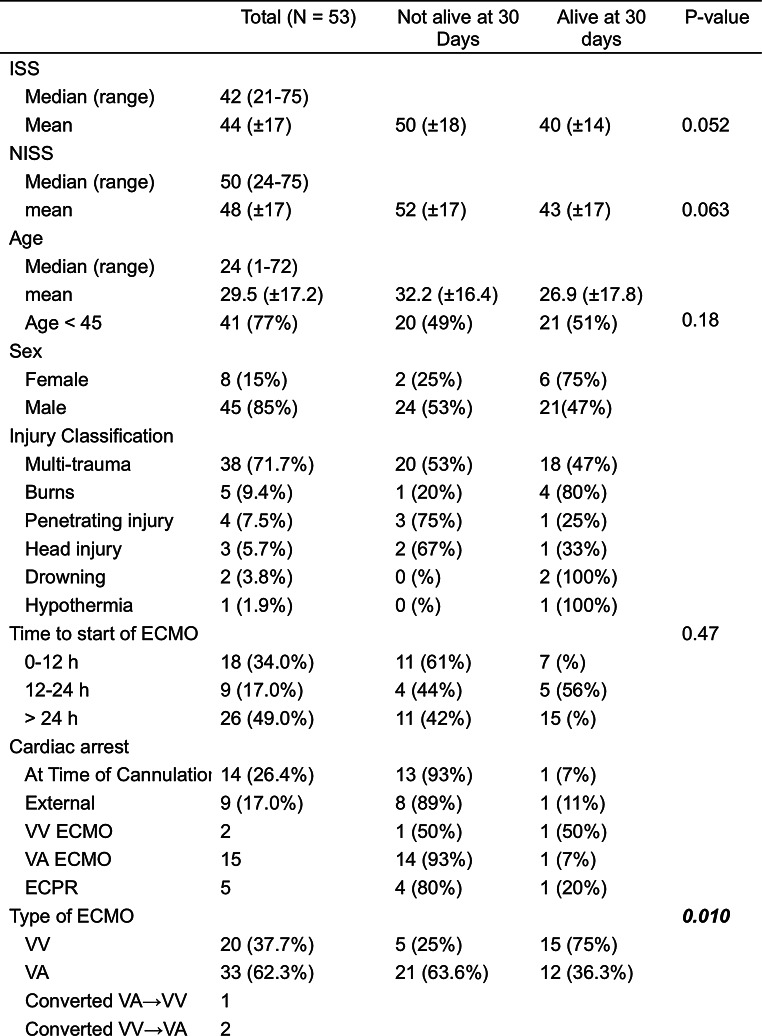
Shows summary statistics of the study population at the time of hospital presentation, prior to ECMO as well as the 30-day survival and mortality

**Table 2:**
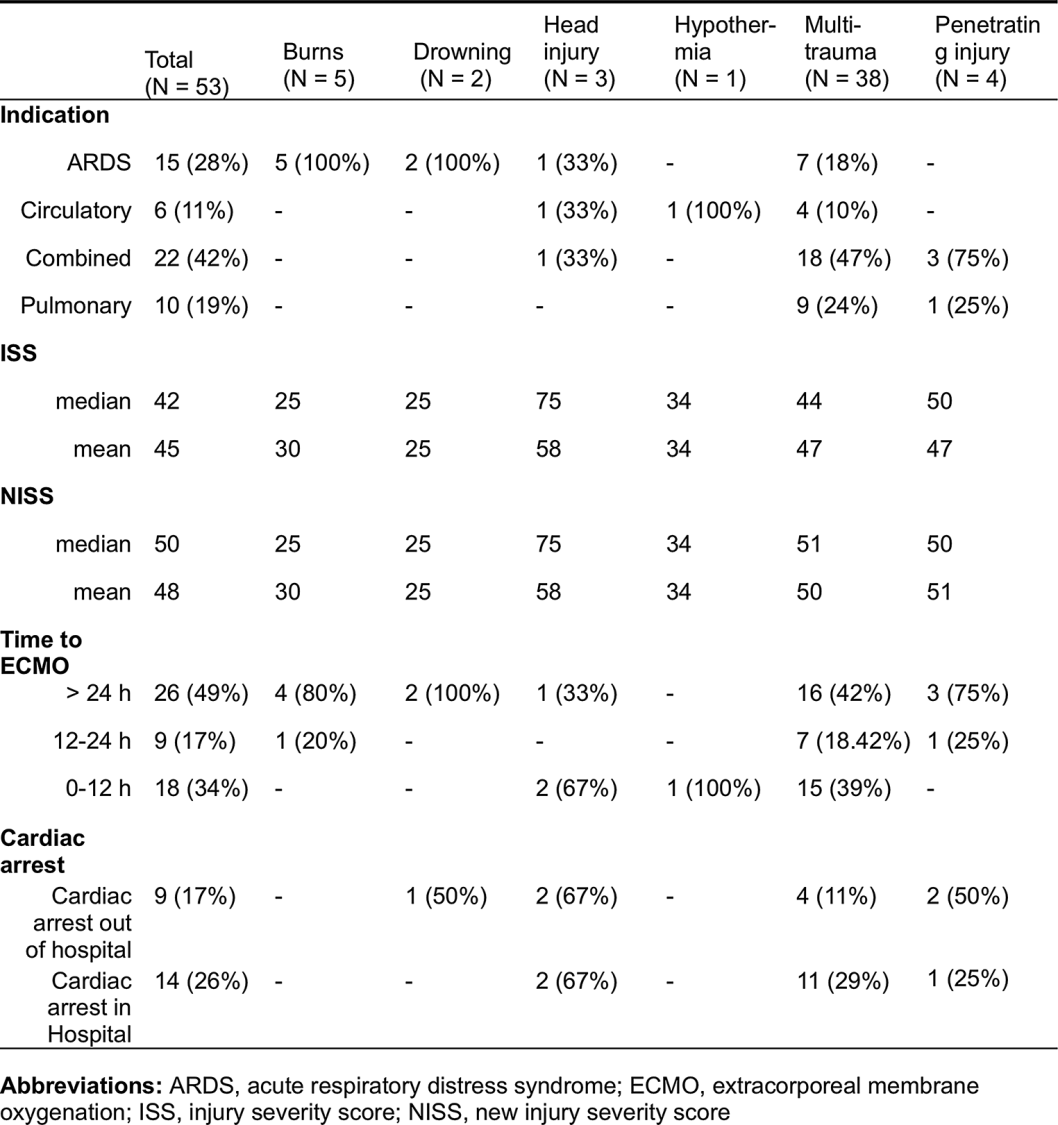
Shows the indications, ISS, NISS, time to ECMO, and number of patients with cardiac arrest for the 6 categories of trauma in the population. The numbers of patients in each group are presented

**Table 3:**
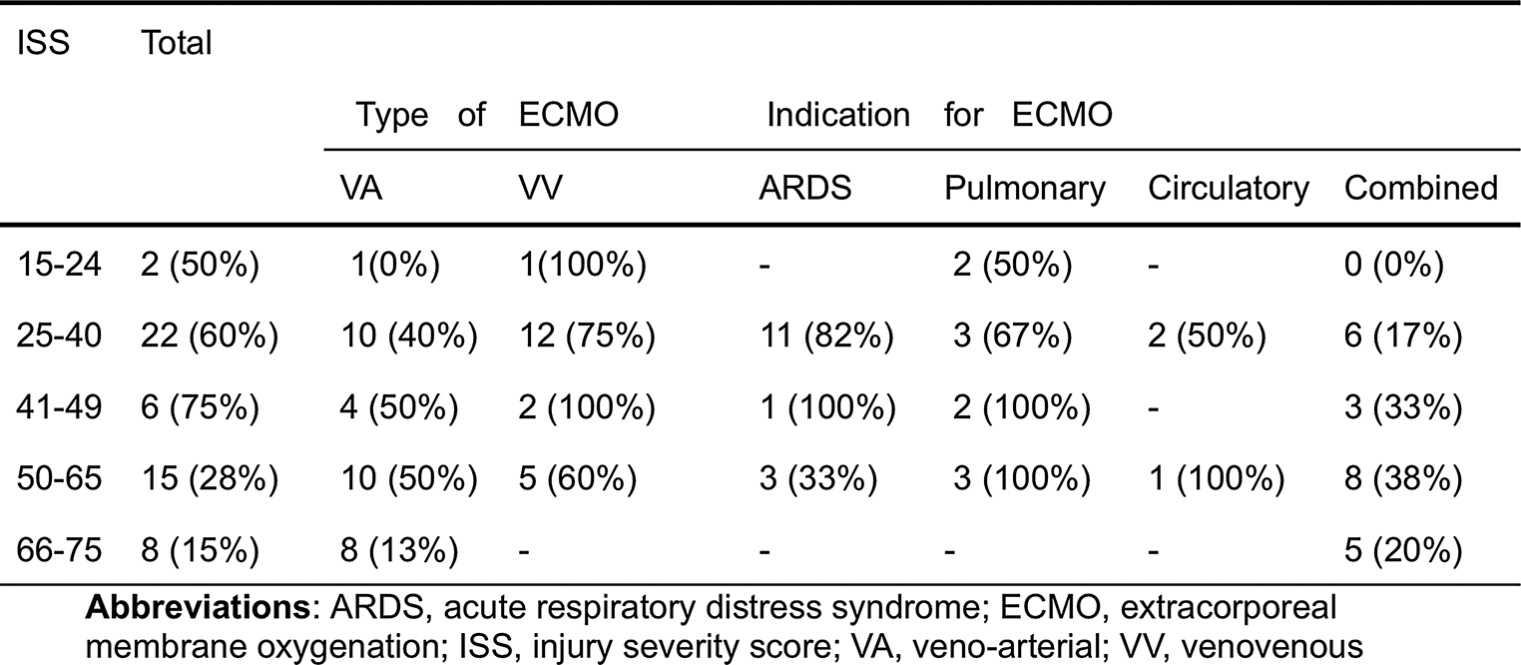
Number of patients in each ISS interval group and 30-day survival (%). The number of individuals within each ISS group is stratified according to mode of ECMO and indication, followed by 30-day survival in parentheses

**Table 4:**
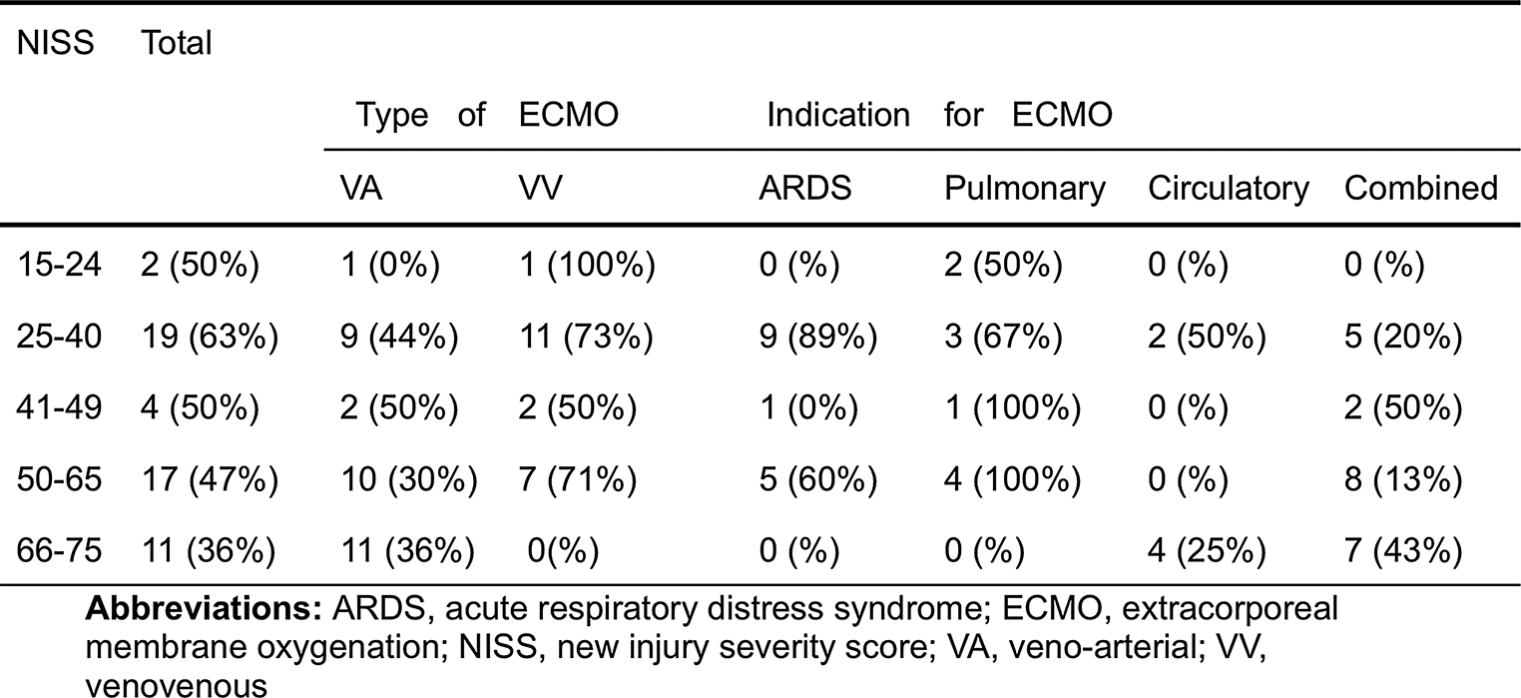
Number of patients in each NISS group and 30-day survival (%). The number of individuals within each NISS group is stratified according to mode of ECMO and indication, followed by 30-day survival in parentheses

### At time of cannulation

The most common indication for ECMO was combined cardiopulmonary failure. Other indications for ECMO and their respective frequencies are listed in Table 5. 51% of the patients were cannulated within 24 h of the trauma, and of these 67% were cannulated between 0 and 12 h post trauma (Table 1). 30% of patients were cannulated at a referring hospital, and 28% were cannulated in-hospital in the emergency room (ER), and the remainder were cannulated in the ICU. Most patients (*n* = 33) were initially treated with VA ECMO, however, of the 20 patients started on VV two were converted to VA ECMO (Table 1). The median time from ER arrival to ECMO start was 36.4 h (5.1-101.5). Time to commencement of VA ECMO was 17.3 h (3.2–65.8), and time to VV ECMO was 60.0 h (34.6-204.4).

**Table 5:**
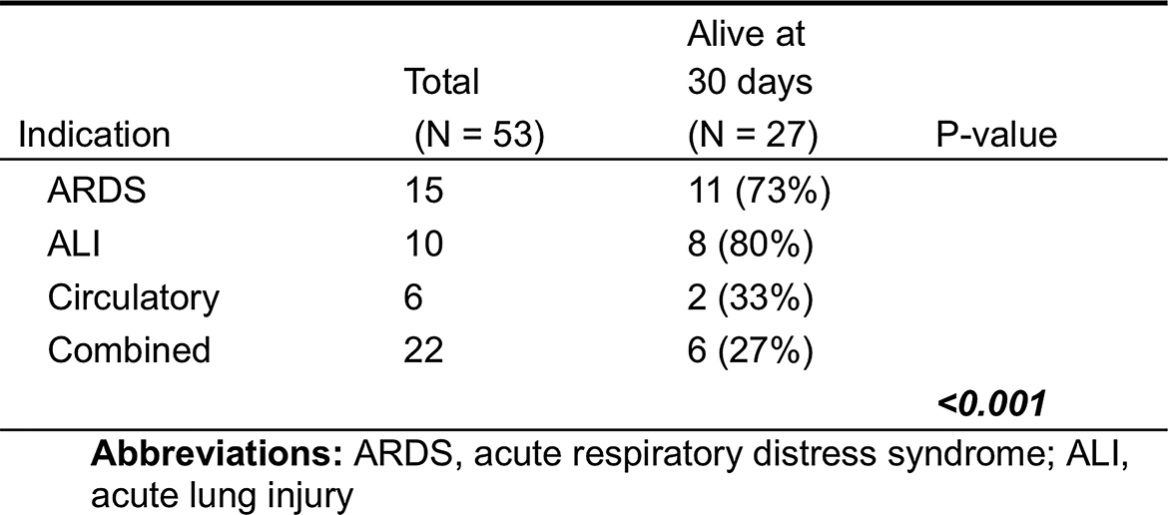
Column one shows the number of patients per indication for ECMO. Column two shows the 30-day survival for each indication, percent survival in parentheses. A p-value was calculated using Fisher’s exact test comparing 30-day survival of patients with ARDS or ALI vs. those with a circulatory or combined pulmo-circulatory failure

### Treatment outcomes

#### General

60% of the patients survived ECMO, and 51% survived to hospital discharge. One patient reportedly died after 13 days due to cerebral infarction suspected to be secondary to ECMO-related coagulopathy. All twenty-seven patients that survived to hospital discharge were still alive 30 days post discharge, and 22 patients are still alive today with the shortest follow-up time of 2 years (median long-term follow-up 13.2 years). Twenty-two patients were cannulated in the ICU, 15 in the ER, and 16 at a referring hospital, with a survival rate of 50%, 27%, and 69%, respectively. There was no difference in 30-day survival over the study period (Fig. 1). Devices used as well as how these may have changed over time can be found in Supplementary Table 1.

#### Injury type

**Fig. 2:**
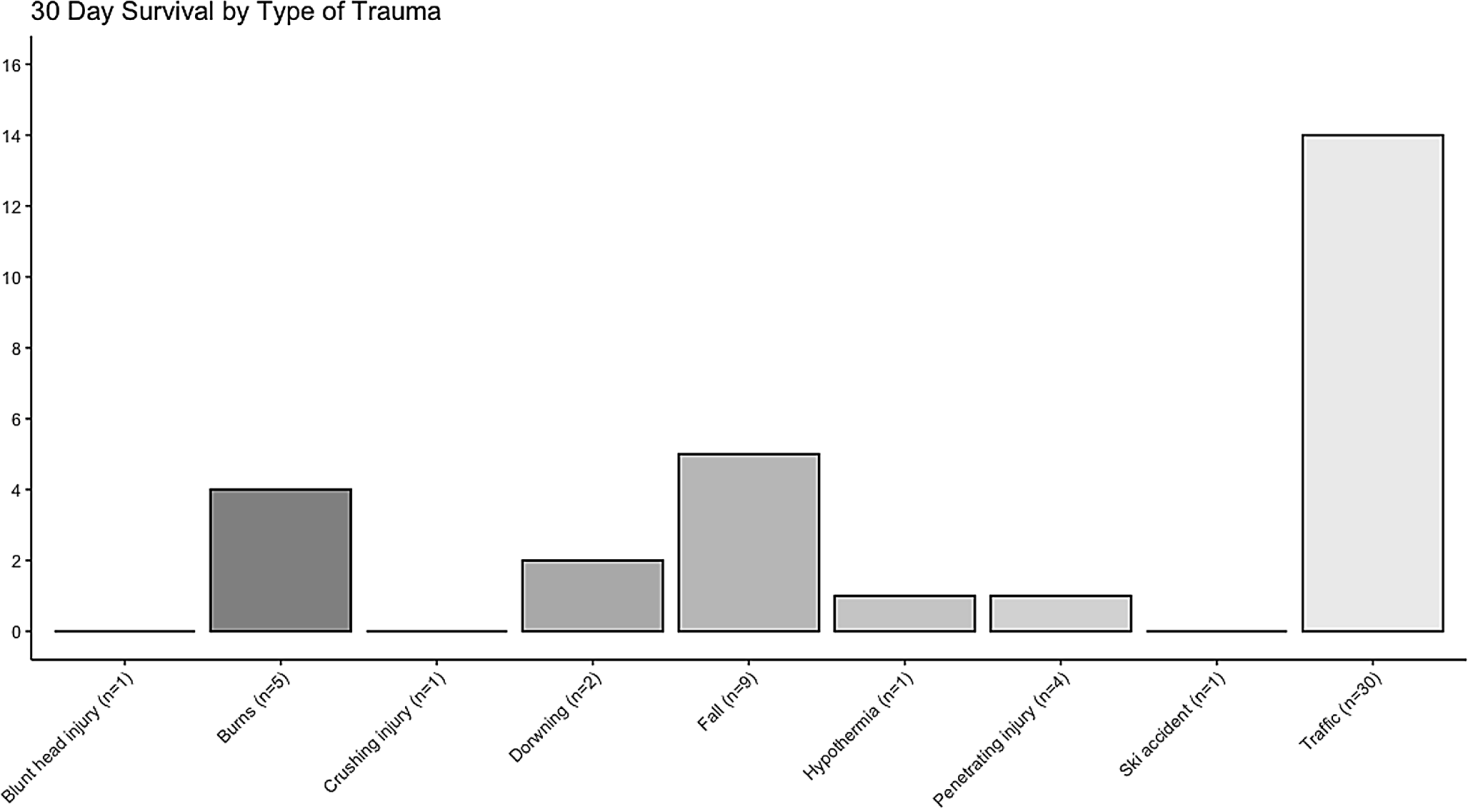
Box-ploy showing the 30 day survival for the type of trauma recorded in this study. Total (n) for each group is presented in parentheses in the x-axis

Thirty-day survival was 48% for traffic accidents, 56% for falls, and 80% for burns (Fig. 2). For those patients where burn severity was quantified (*n* = 2), both were classified as severe burns (> 40% of body surface area) [27]. The patients were also stratified based on type of injury, as shown in Table 1. The most common form of injury was multitrauma with a survival rate of 47%. Survival was substantially higher for burns, drowning, and hypothermia and lower for penetrating injuries and isolated head injuries (see supplementary Fig. 1). However, 58% (*n* = 22) of the multi-trauma patients had a concomitant head injury. Hence, for all patients with a head injury, 30-day survival was 54% (*n* = 14/26). Lastly, there were five confirmed suicide attempts of which three (60%) survived 30 days post discharge.

#### Survival outcomes based on ECMO indication

The patients who were treated with ECMO due to ALI had the highest 30-day survival rate (80%), followed by ARDS (73%). There was a significant difference between patients initiated on ECMO due to ARDS or ALI compared to patients who received ECMO due to circulatory failure or combined circulatory and pulmonary failure (*p* < 0.001). Patients with combined circulatory and pulmonary failure had the lowest survival outcome, in all six patients representing a survival rate of 27% (Table 5).

#### Cardiac arrest

In total, 17 patients experienced cardiac arrest (CA) before or at the time of cannulation. Nine patients arrested outside of the hospital. Of these, eight had a return of spontaneous circulation (ROSC). Fourteen patients had ongoing cardiopulmonary resuscitation (CPR) during the cannulation procedure, of which six had CA outside of the hospital. Fifteen of 17 patients were placed on VA ECMO and two on VV ECMO. One VA and one VV patient survived. Extracorporeal cardiopulmonary resuscitation (ECPR) was used in five cases with only one surviving 30 days post discharge.

#### Complications to trauma

Overall, 77% (*n* = 41) of the patients suffered some form of complication, and 60% experienced two or more complications in addition to the trauma per se. The most common complication was severe neurological disability, followed by sepsis and hemorrhagic shock. The highest morbidity was associated with severe neurological disabilities, whereas sepsis showed the highest 30-day survival (55%). Complications and associated survival rates are listed in Table 6.

**Table 6:**
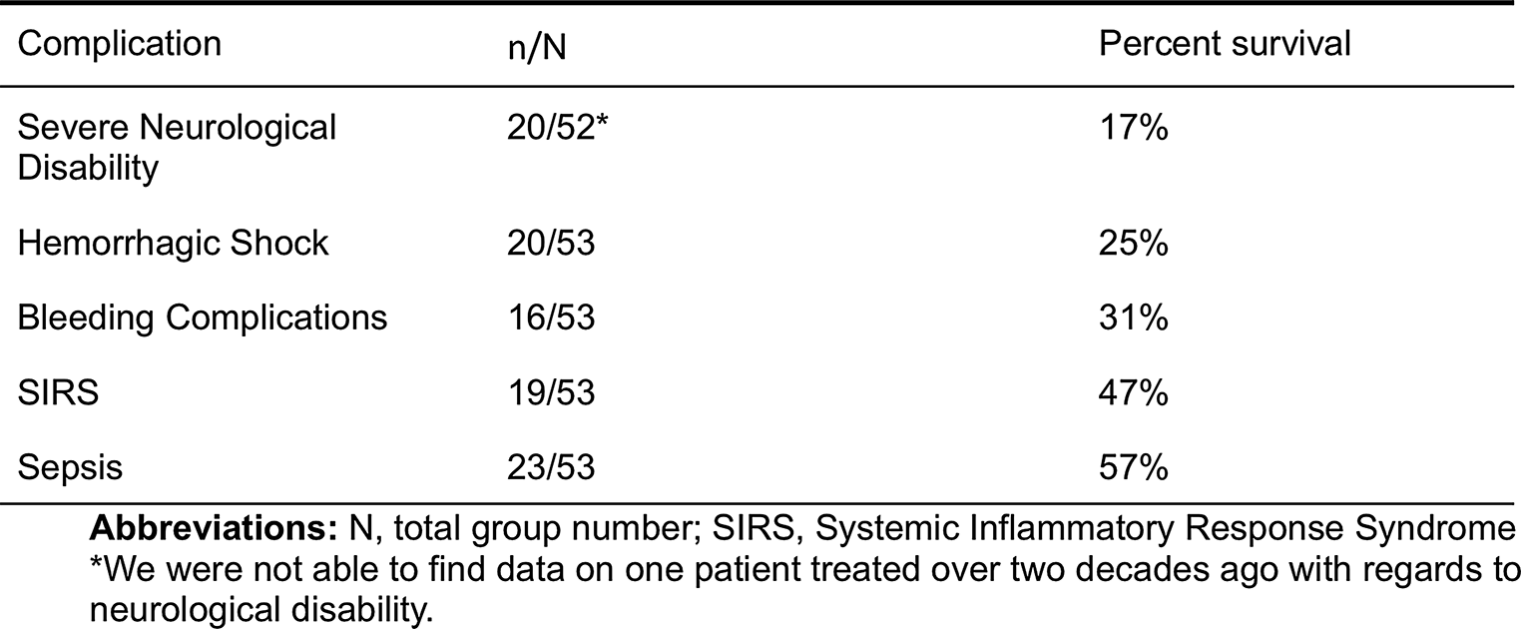
Shows the frequency of complications (n) during hospital stay as well as the 30-day survival in percent for each complication

#### Survival outcomes and trauma scoring

**Fig. 3:**
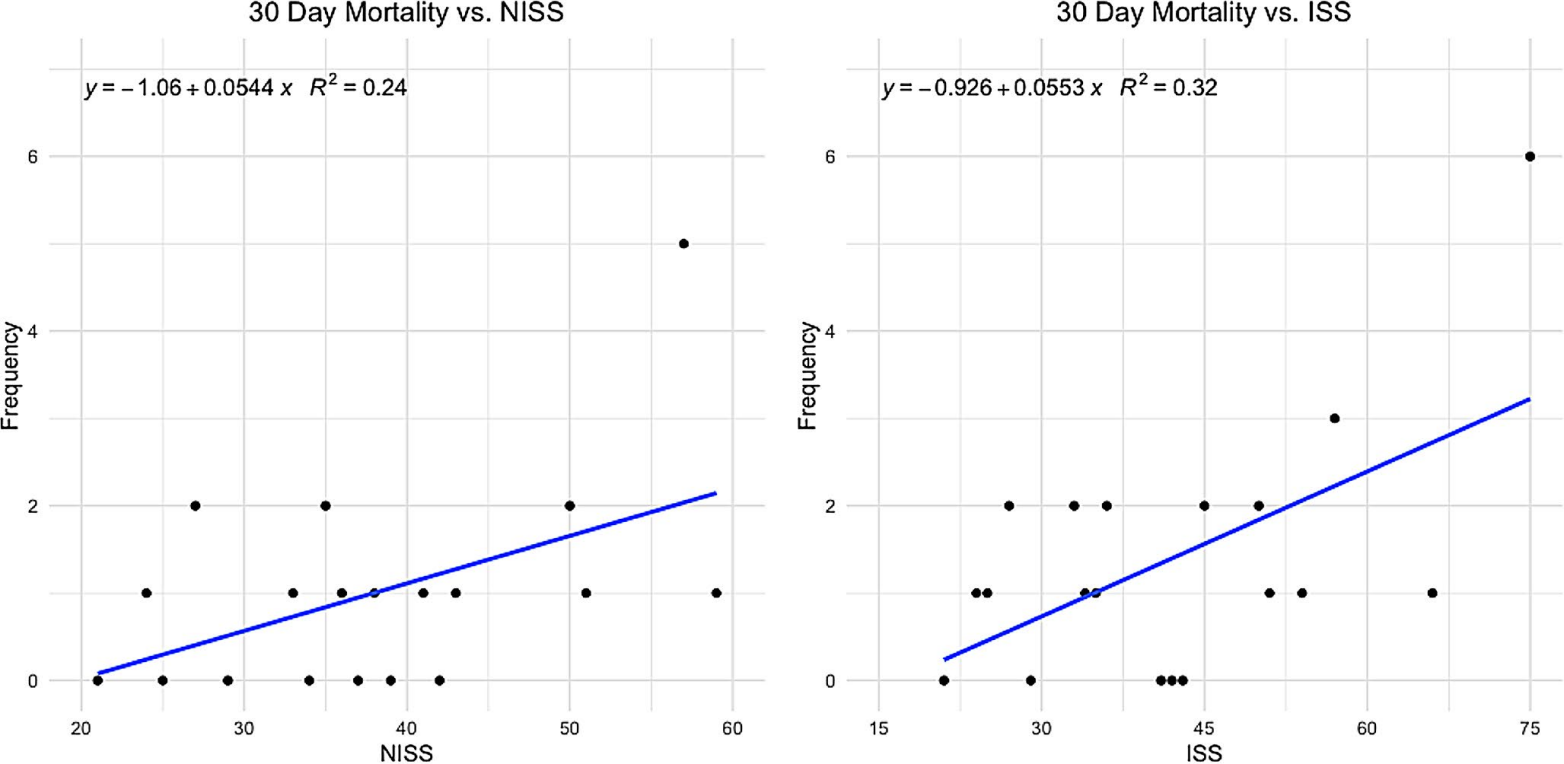
Scatterplot showing 30-day mortality vs. NISS and ISS, respectively. A linear regression model is shown for each

**Fig. 4:**
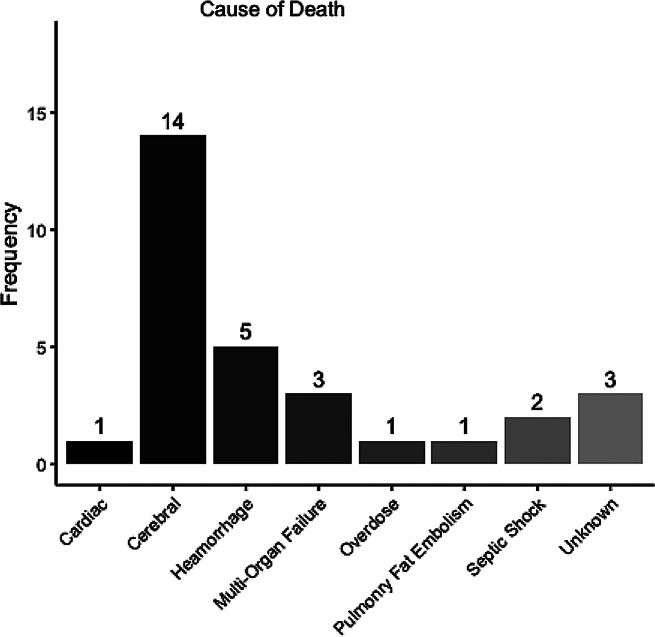
Box plot of the frequency of causes of death

A scatter plot of NISS and ISS vs. mortality within the first 30 days showed a correlation of higher mortality in patients with higher NISS (Fig. 3). The ISS score in survivors after 30 days was similar to the deceased (36 IQR:25–51 vs. 50 IQR:34–64, *p* = 0.052) (Table 1). This relationship was repeated for NISS. Likewise, no difference was observed concerning age or time from trauma to start of ECMO. Patients supported with VA ECMO had a 30-day survival of 34% compared to 76% in the VV group (*p* < 0.005). The seventeen patients cannulated at a referring hospital and then transported to our unit on ECMO showed a survival rate of 69% compared to 57% in those cannulated in-house (*p* = 0.14) (Table 1). Concerning complications, massive hemorrhage as a presenting symptom was associated with poor outcome (*p* < 0.01). Five out of 20 patients with reported bleeding survived. Overall, 26 patients died before or within 30 days of discharge (causes of death, see Fig. 4).

## Discussion

In this retrospective analysis we present data on 30-day survival in 53 patients treated with ECMO at a Level 1 trauma center. Our data shows significantly higher survival patients treated with VV than with VA ECMO. There was also a significant difference in survival based on the indication for ECMO support, and no difference was observed among patients cannulated at a referring hospital and then transferred to our hospital compared to those cannulated in-house. Furthermore, we found no difference in survival outcomes throughout the study period. According to the ELSO Registry, overall survival for adult ECMO patients in North America and Europe over the last 5 years is 53% [28]. This is comparable to our results of 51%, and results previously reported by others [29–30]. Hence, our results provide further evidence that ECMO can be used successfully to support patients with very severe trauma, in particular, patients with primary lung pathologies.

Major trauma is the primary cause of death among young adults. The use of ECMO in trauma, while still controversial, has become more prevalent over the past decades. However, most reports are case studies or retrospective analyses with a limited number of patients, usually less than 20 subjects [29]. With the increased interest in ECMO for trauma there is a need for clearer indications to identify the patients most likely to benefit from this support.Trauma patients are heterogeneous both with respect to comorbidities, age and mechanism of injury. The mechanical impact of a motor vehicle accident has a different impact from a stab wound or a drowning but the common denominator is the problem of oxygen delivery, for which ECMO provides an unique approach.

To quantify the severity of trauma in this patient population we chose the widely used ISS and NISS for easy comparison to previous studies. Our sample population, although small, was similar to the trauma population in Sweden with respect to mechanism of injury with the most common cause being traffic accidents (ca. 50%) followed by falls. However, the sex distribution in our sample was more heavily skewed towards males than that which is reported by the Swedish trauma registry (i.e. roughly two thirds). Furthermore, our sample was comparatively young with a median age of 24 compared to 37 for the trauma population at large [31]. Furthermore, the trauma severity in our population was quite high. The mean NISS and ISS assessed in this study were 46 and 44, respectively, thus representing a very high risk of mortality as shown by Copes et al., and Ghorbani [11, 32]. Overall, 41% of the patients had an ISS greater than 50, which otherwise was associated with almost 100% mortality rate [11]. According to the Swedish trauma registry roughly 12,000 patients are brought to hospital due to trauma per year, of which about 2–3% have an NISS > 40 [31]. Even though ECMO is not a typical supportive modality in the trauma patient, the results showed more than half of the patients were still alive at follow-up. Furthermore, we divided both the ISS and NISS into intervals according to Copes et al. showing that there was similar distribution with a slight tendency towards the higher bins within the VA group, but with a higher proportion 30-day survival for the patients on VV ECMO (Tables 3 and 4). The cause of higher mortality in the VA group might be partially explained by the fact that most patients with cardiac arrest and ongoing CPR, a subgroup with a very high mortality rate, were placed on VA ECMO.

Almost two thirds of the patients were placed on VA ECMO in contrast to earlier studies where pure respiratory support was more common due to ALI/ARDS [29–30]. The ELSO Registry reported an overall survival rate of 43% for all VA ECMO patients, and 30% for patients treated with extracorporeal cardiopulmonary resuscitation [28]. Previous studies on the use of VV ECMO in the trauma population show a survival range of to 56–89% compared to 42–63% for VA ECMO [29]. This was similar to our findings of 75% 30-day survival in the VV ECMO group, and 36% for VA ECMO. This difference may be explained by physiological differences in the patients, whereby patients requiring VA ECMO had severe hemorrhagic shock, cardiac arrest, cardiac failure or a combined cardio-pumlmonary failure secondary to injuries. Trauma patients in cardiac arrest had a particularly poor survival outcome, and after the exclusion of the patients with CA, survival from VA ECMO was 61% as compared to 75% on VV ECMO. Taken together, this may reflect that trauma patients who present with lung problems, and who are then placed on VV ECMO are more likely to benefit from ECMO support.

ECMO support increases the risk of bleeding complications and subsequently the risk of cerebral hemorrhage [33]. Hence, most clinicians may feel apprehensive to offer ECMO to trauma patients with head injuries. In this study, most of the patients were multi-trauma, and nearly half presented with head injuries. Three patients were categorized as isolated head injuries, which is a relative contraindication for ECMO. Despite this, one of these patients survived. Of the 26 patients with head trauma, 54% survived 30 days after discharge, a survival rate, similar to the overall survival rate for the study population (51%). Previous studies have shown benefits in patients with traumatic brain injury (TBI), both from VV and VA ECMO [34–35]. Considering the patients with TBI in both this study and previous studies had an outcome comparable to the typical trauma patient supported on ECMO, the risk-benefit of ECMO use in patients with TBI should be re-evaluated. ECMO can safely be used for several days without anticoagulation, particularly in the coagulopathic trauma patient, and can therefore be considered in patients with massive hemorrhage and TBI [36].

Another area of limited knowledge is the outcome of trauma patients transported on ECMO. We observed no difference in mortality between those patients cannulated and then transported on ECMO compared to those who were cannulated in house. The mobile ECMO team in this study has extensive experience including both in-hospital cannulations and multiple ECMO transports of non-trauma patients, and has only recorded three deaths during transport over a 30-year period, of which one was a trauma patient [37–39]. The analysis showed that cannulations at referring hospitals were generally performed at a later stage (> 12 h post trauma), and the patients still alive had been stabilized by local intensive care for several hours and thus more stable than the patients brought directly to our university hospital from the trauma site. This finding may be subject to selection bias influenced by more strict selection criteria where the time factor and prognosis would strongly impact a decision to dispatch the mobile ECMO team. Nonetheless, the high survival rate (69%) suggests that with strict selection criteria a mobile ECMO team that transports patients to a larger ECMO center may be a life-saving strategy for patients suffering from severe trauma.

### Limitations

Limitations of this work include the sample size, which was relatively low from a general perspective but rather high given the targeted ECMO population from a single center. The applicability of findings from a high-volume center may not extend to institutions with less experience, potentially limiting their generalizability. Our ECMO management has developed over the years as a slow continuous process based on our own experiences and influences from international collaborations, others’ experiences, and research. Our unit does not manage ECMO according to any preset treatment protocol since extracorporeal life support (ECLS) is what we do every day being one of extremely few intensive care units (ICU) worldwide dedicated exclusively to ECLS. This fact brings a limitation regarding generalizability of the results from this study. Other limitations may be patient heterogeneity, mechanism of injury and age (1–72 years). Further limitations include the time period of the study, 22 years, during which changes in practice occurred, and that patients recovered from other hospitals may have been subjects of selection bias. The key strengths of this study were the Swedish Civic registration number, which allowed for long-term follow-up of patients, and the local databases for trauma and ECMO support.

## Conclusions

This single-center retrospective study indicated that ECMO support in patients with severe to very severe trauma may benefit from ECMO in terms of survival. The data suggests benefit of out-reach services by a mobile ECMO team to cannulate and transport the patient to a high-volume ECMO and trauma center. Further multicenter studies with larger patient numbers are needed to assess the benefits of ECMO in the trauma setting.

## Electronic supplementary material

Below is the link to the electronic supplementary material.

Supplementary Material 1

Supplementary Material 2

## Data Availability

No datasets were generated or analysed during the current study.
