# Supplementary figures and images for: Extracorporeal membrane oxygenation in trauma: a single-center retrospective observational study
Source: Eur J Trauma Emerg Surg. 2025 Jan 27;51(1):88. doi: 10.1007/s00068-024-02734-1 (PMC11772380; doi:10.1007/s00068-024-02734-1)

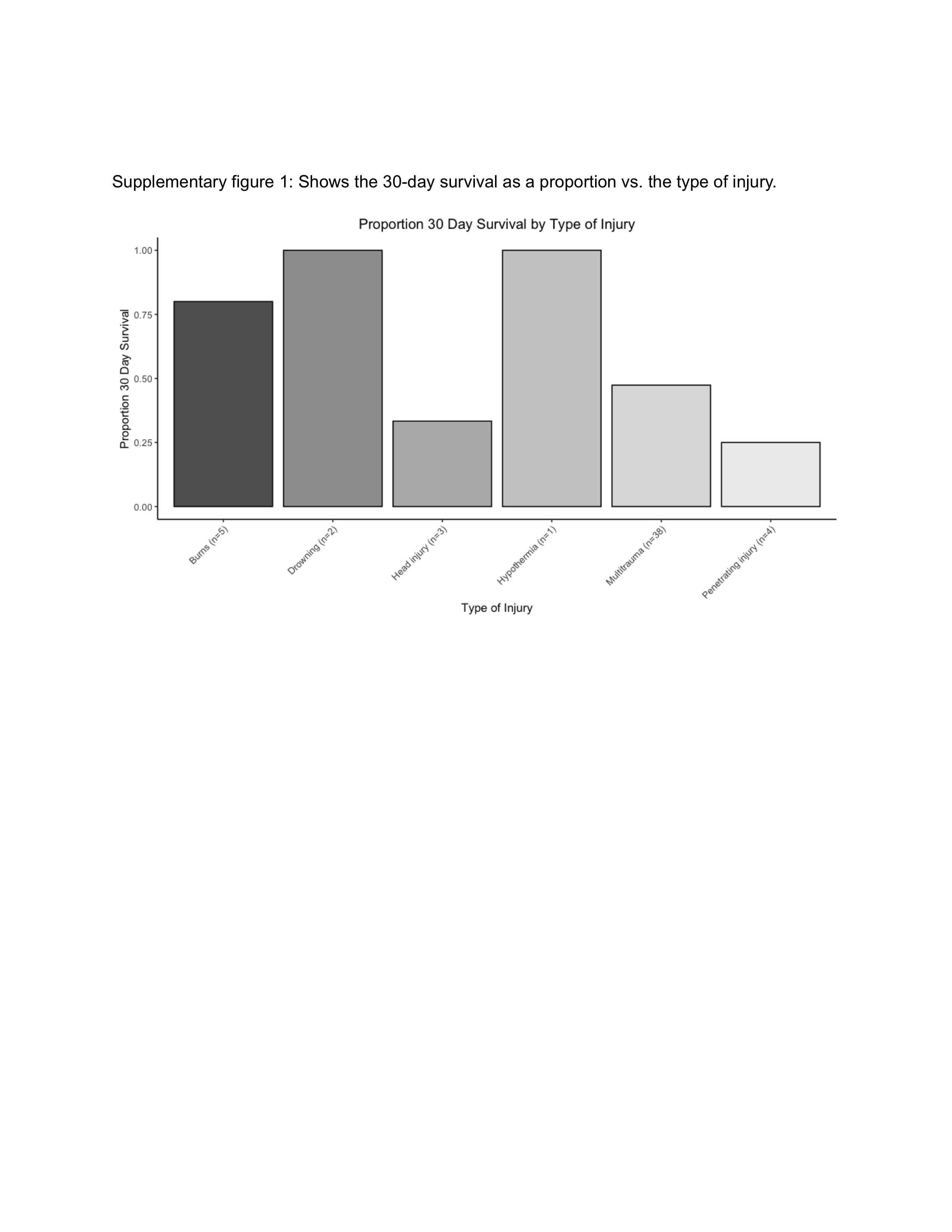

Supplement: Supplementary file 1 — Supplementary Material 1 [file 68_2024_2734_MOESM1_ESM.tiff]
